# Progress on the Congo

**Published:** 1891-11

**Authors:** 


### PROGRESS ON THE CONGO.

White sojourners in the Congo country are themselves astonished at the number and importance of the measures now under way for the protection of the many thousands of natives against murder and rapine, and to spread order and civilization throughout the great river basin. Every mail from Europe brings news of fresh edicts to be enforced, of new police or educational measures to be carried out, of new steamboats coming or new expeditions projected. The best of it is that the natives are beginning to grasp the idea that all this conduces to their material advantage, safety and welfare, and they are, therefore, becoming more amenable to discipline and law. The State is dealing with them as with the Arabs, gently if possible, but is employing severer means, if necessary, to enforce obedience and respect.

The directions in which the Congo State is now chiefly working is for the suppression of the liquor traffic, the extinction of murderous slaves' raids, the opening of new routes of traffic, and the drilling of the many hundreds of natives employed by the government in some features of military tactics, and in industrial pursuits.

All philanthropists will feel grateful to the Congo State for its new edict strictly prohibiting the sale of all alcoholic liquors in all that part of the great river basin lying east of the Inkissi River. This river is forty miles west of Leopoldville, and the country between it and the Atlantic Ocean has been so long occupied by white liquor traders that it will not be possible, all at once, to carry out drastic measures for the suppression of the traffic. The trade, however, is to be strictly regulated, and its volume will be diminished, if possible, by taxes imposed upon the dealers.

The Congo State is thus showing itself to be a most beneficent and effective agent in the protection of the people against one of the greatest evils that threaten them.

The State has at last succeeded in hemming in the Arab dealers on the west and north, limiting their further raids down the Congo and along its branches. They have done this by establishing the seven military stations on the north and south tributaries of the Congo occupied by well armed forces, and frequently visited by the trading vessels and gunboats of the State. The natives have learned that these posts are places of refuge, where, under the blue flag of the Congo State, they may find safety from Arab murderers or oppressors of their own tribes. The Arabs understand that the limits of their slave and ivory hunting fields have been reached, and they are submitting as gracefully as possible to the inevitable. Everywhere throughout the growing regions which the Congo State is bringing under its influence the horrid custom of human sacrifices is beginning to be severely punished.

As an instance of the work the State is doing may be mentioned the progress of Bangala station, where the State is undertaking to educate and care for 170 children of its black employees. It is teaching them to read. It houses them in well ventilated and well built huts. It gives them their meals in a large dining shed, where an immense table flanked by benches gives accommodation to the entire little community at once. Many men and women are employed on the farm, about three-quarters of a mile square, raising the food for these children and the station employees, and three cooks are employed solely in preparing the meals for the little wards of the State.

A large hospital has been built behind the station, with accommodations for forty patients, besides chambers for the convalescent. At present the hospital contains twelve blacks, including eight children, three men, and one woman, out of the population of 500 souls who form the black personnel of the station.

A number of the natives are employed in the manufacture of brick, and at present two large furnaces are burning 120,000 brick each.

The construction of the Congo Railroad is going on rapidly. The survey has been completed throughout the first twenty-five miles of the road, where all the engineering difficulties are accumulated. The grading of the road has been completed for nearly four miles. There are at present ninety-eight white engineers, agents, and workmen engaged on the road, besides a thousand native workmen drawn from many parts of Africa. The last mail from Europe reports that three locomotives and quite a number of flat cars are constructing, and will soon be shipped to Matadi, the starting point of the railroad. The health of the white force is very good, and they are carrying on the work with a great deal of enthusiasm.

In all directions it may be said that the work of the Congo State, though beset with many difficulties, is making favorable progress, and the white men on the ground, almost discouraged as they are at times by the immensity of the work before them, are still surprised themselves when they see the large amount that has been done and the rapidly widening opportunities for making still greater progress.

